# Synthesis and characterization of novel functional hyperbranched polyamides from AB_2_ units: effect of extra functional groups

**DOI:** 10.1080/15685551.2020.1790727

**Published:** 2020-07-09

**Authors:** Hamidreza Rezania

**Affiliations:** Department of Organic Chemistry, Faculty of Chemistry, Kharazmi University, Tehran, Iran

**Keywords:** Novel AB_2_ monomers, hyperbranched polymers, polyamide, functional groups

## Abstract

Hyperbranched polymers (HPs), which in terms of structure may be compared to the branching structure of trees, are referred to as tree-like materials, but role of leave in these tree-like polymers is neglected and much attention has only been paid to their branches. In fact, functional groups in these polymers play a vital role the same as the role of leaves in trees. Therefore, in this paper, an attempt has been made to design and synthesize three AB_2_ monomers containing extra hydroxyl and nitro groups. The benefits of their presence in the structure of produced hyperbranched polyamides (HPs) are investigated. The polymer structure was characterized by FT-IR and ^1^ H NMR. The solubility of synthesized HPs was studied in different protic and aprotic solvents. The thermal stability of the prepared HPs was investigated by thermogravimetric and differential scanning calorimetric analyses. The photoluminescent properties of the HPs were also investigated.

## 1. Introduction

Some special and interesting features of hyperbranched polymers (HPs), such as the ease of preparation, low melt and solution viscosity, high solubility, globular structure, abundant functional groups, and excellent thermal properties make them useful in many applications, including nanotechnology [1], coatings [2], medical and biological applications [3]. HPs are frequently used as blends with many copolymers [4]. Properties of such blends can be improved depending on different structures of HPs. HPs, due to enormous number of functional groups, are often employed as the core of core–shell block copolymers [5,6]. Importantly, the number of terminal groups in a hyperbranched polymer is a crucial index in drug conjugation [7], polymer grafting [8], and catalyst supporting [9].

Aromatic polyamides (aramids), because of exceptional chemical resistance, high thermal stability, excellent mechanical properties, and low flammability are accepted as high-performance engineering polymers [10,11]. However, high melting/softening temperature, poor solubility, high stiffness, and high crystallinity have resulted in a number of difficulties in the processing of these polymers [12]. One of the famous methods to improve the solubility and processability of polymers is branching. Highly branched polymers may be prepared by different strategies, particularly polycondensation of an AB_2_ monomer, which is cheap and convenient and has attracted great attention [13,14]. Moreover, some HPs have been synthesized via the A_2_ + B_3_ synthetic method [15,16].

**Figure 1.**
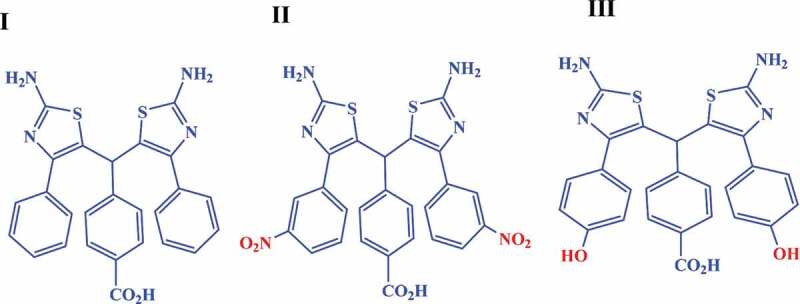
Structure of three new AB_2_ monomers synthesized in this study

Some researchers [17,18] have introduced HPs as tree-like materials, while HPs with no functional groups are resemble to leafless trees which only contain stems and branches. Leaves of a tree provide a lot of benefits to man, plants, and ecosystems, such as, oxygen production, removing excess carbon dioxide, decreasing the temperature, food production (sugar which is stored in fruits, seeds and tuber (potatoes) of plants), medicine, fiber, dyes, beauty of places, etc. As mentioned, leaves give trees tremendous benefits. What plays the role of leaves in HPs is free functional group. Functional groups improve solubility and processablility, and provide reaction sites and in general develop performance of the final polymers. In fact, the production of HPs with different functional groups can make them suitable for various applications [19]. The presence of different terminal functional groups and extra substituent functional groups like hydroxyl and nitro groups can change the polymer properties and provide useful scaffolds or supports for additional modification of materials. For example, free hydroxyl groups in hyperbranched polyethylene glycols can be employed to design hydrogels for biomedical uses (tissue engineering drug delivery, protein immobilization, bioconjugation with peptides, etc.) [20]. There are no any studies on the preparation of HPs with extra functional groups, and most of the studies are centered on the synthesis of different kinds of HPs. Regarding the above-mentioned points, three new AB_2_ monomers (Figure 1) with and without extra functional groups were synthesized and employed for the preparation of three novel hyperbranched polyamides (HPs) with nitro and hydroxyl groups. The prepared polymers were characterized by NMR and FTIR. The thermal properties, solubility behavior, and fluorescence characteristic of the resultant polymers were investigated to understand their structure–property relationship. The prepared HPs seem to be very promising for the future applications in thin-film composite nanofiltration membrane and ultrafiltration membranes.

## 2. Experimental

### 2.1. Materials

Acethophenone, diiodine, thiourea, 4-hydroxyacetophenone, 4-formylbenzoic acid, 3-nitroacetophenone and 4′-hydroxyacetophenone were obtained from Sigma-Aldrich Co., Germany. Pyridine (Py, Fluka) and N-methyl-2-pyrrolidone (NMP, Merck) as polymerization solvents were purified by distillation under reduced pressure over calcium hydride (CaH_2_). Lithium chloride (LiCl), triphenyl phosphite (TPP), *N, N*-dimethylacetamide (DMAc), and other solvents such as dimethylformamide (DMF), dimethyl sulfoxide (DMSO) and tetrahydrofuran (THF) were used as received from Merck company.

### 2.2. Synthesis of diamine monomers

**Figure 2.**
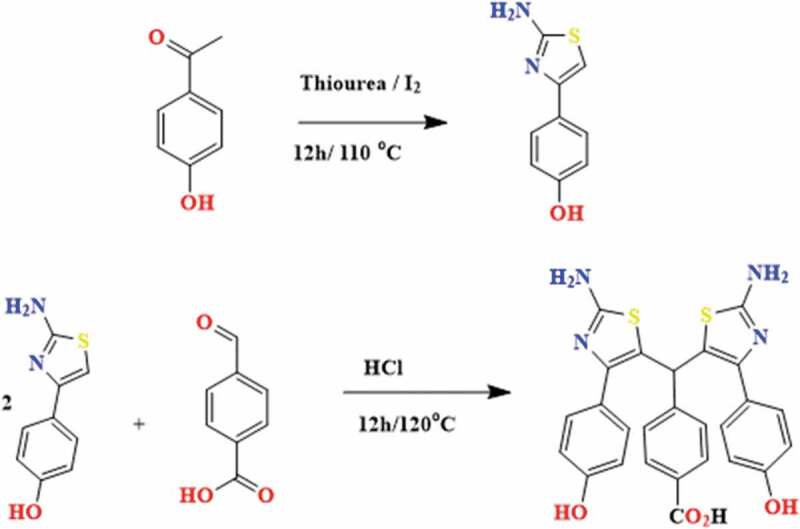
Two-step synthesis of AB_2_ novel monomers (hydroxy-containing monomer (III))

Monoamines (MA) were prepared using the reported method [21]. Briefly, a mixture of 1.53 g (20 mmol) of thiourea, 1.36 g (10 mmol) of acethophenone derivative (here 3-nitroacetophenone), and 2.53 g (10 mmol) of diiodine were stirred and heated to melt at 110°C for 12 h. After the reaction media became solid, the mixture was dissolved in 200 mL water with heating. The obtained solution was filtered and neutralized with a basic solution (sodium hydroxide 1 M) to precipitate a pale yellow solid. The obtained precipitate was filtered, washed with water, and recrystallized from H_2_O/EtOH (1:1) to afford 4-(3-nitrophenyl) thiazol-2-amine (yield = 84%). To prepare other monoamines, the same procedure was followed but, in this case, acethophenone and 4-hydroxyacetophenone were used as starting materials (Figure 2).

To prepare nitro-containing monomer (II), a mixture of 4-formylbenzoic acid (1.51 g, 10 mmol), 4-(3-nitrophenyl) thiazol-2-amine (3.84 g, 20 mmol), and hydrochloric acid (50 mL) was prepared in a 100-mL round-bottom flask and refluxed for 12 h at 110°C. Then the reaction mixture was poured into 200 mL of water and neutralized with NaOH. The obtained product was filtered and dried (Figure 2) [22].

### 2.3. Synthetic procedure of hyperbranched polyamides (HPs)

**Figure 3.**
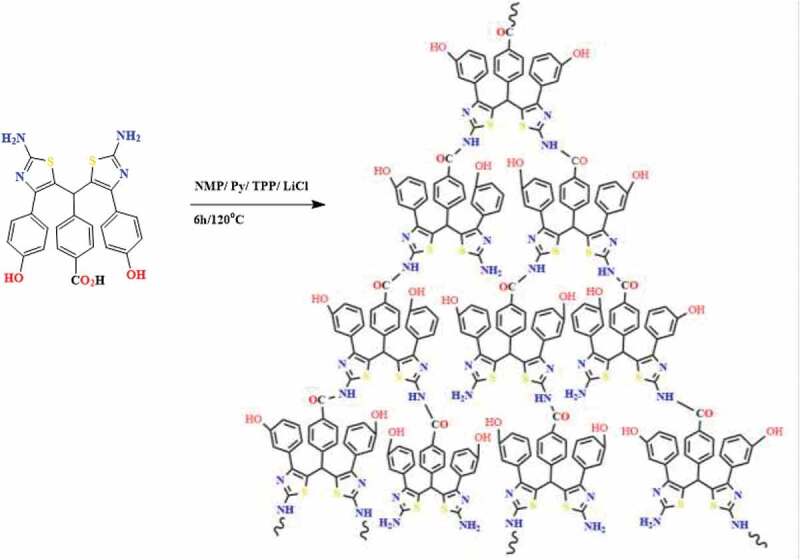
Synthesis of hyperbranched polymers (HP3 is shown as a typical model)

To prepare all hyperbranched structures, the Yamazaki method was employed [23]. A mixture of 10 mmol of AB_2_ monomers, 3 mL of NMP, 2 g of lithium chloride, 3 mL of TPP and 2 mL of pyridine was refluxed for 6 h at 120°C (Figure 3). Then the reaction media was cooled and poured into 100 mL of methanol. After 3 h stirring in methanol, the product was filtered and washed carefully with hot methanol and water and dried under vacuum [24]. HP1, HP2, and HP3 were employed to show the HPs obtained from I, II and III monomers, respectively.

### 2.4. Characterization of polymers

To study the functional groups of the compounds, the Fourier transform infrared (FT-IR) spectra were measured by means of a Perkin Elmer FT spectrum RX1 (USA). To investigate the inherent viscosities (η_inh_) of synthesized polymers, a solution of 0.5 g dL^−1^ of HPs in DMAc was prepared, and then an Ubbelohde suspended-level viscometer was used to measure their viscosities at 30°C.

The monomer and polymer structures were studied by recording their solution state ^1^ H NMR (300 MHz) and ^13^ C NMR (75 MHz) spectra on a Bruker DRX 300 AVANCE spectrometer (Germany) using deuterated dimethyl sulfoxide and trimethylsilane (TMS) as solvent and reference, respectively.

A Du Pont 2000 thermal analysis system (Mettler Toledo-Switzerland) was used for thermo-gravimetric analysis (TGA) under N_2_ atmospheres at a heating rate of 10°C min^−1^. Glass transition temperatures (T_g_) were investigated by a differential scanning calorimetry (DSC-2010 model) thermal analysis (Mettler Toledo-Switzerland) apparatus at a heating rate of 10°C min^–1^. A Shimadzu RF-1501 spectrofluorophotometer was used to obtain the fluorescence spectra.

## 3. Results and discussion

### 3.1. Characterization of monomer

**Figure 4.**
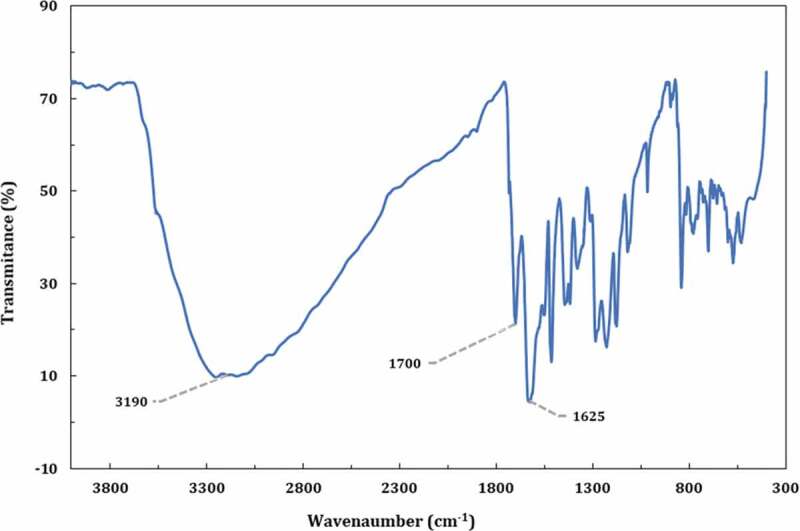
FTIR spectrum of monomer III

The solubility and processability of aramids can be improved by branching the polymer chain and introduction of some hydrophilic groups into the chains. Therefore, in this work, an attempt has been made to simultaneously introduce branching and functional groups including hydroxyl and nitro groups into polyamides to improve their properties (Figure 3). The AB_2_ monomers were synthesized in two steps, which the synthesis route is depicted in Figure 2. The FTIR, ^1^ H NMR and ^13^ C NMR results confirmed the formation of AB_2_ monomers structures. As a sample, the FTIR spectrum of monomer III is shown in Figure 4. The distinctive absorption bands of amine, hydroxyl and carboxylic acid groups are observed in the FTIR spectrum of monomer III as a mixed broad band at 2400–3400 cm^−1^. As seen, the carboxylic acid C = O stretch has appeared at 1700 cm^−1^. Moreover, it shows a distinct absorption band as mixture at 1625 cm^–1^ related to the N-H bending and -C = N- stretching of thiazole.

**Figure 5.**
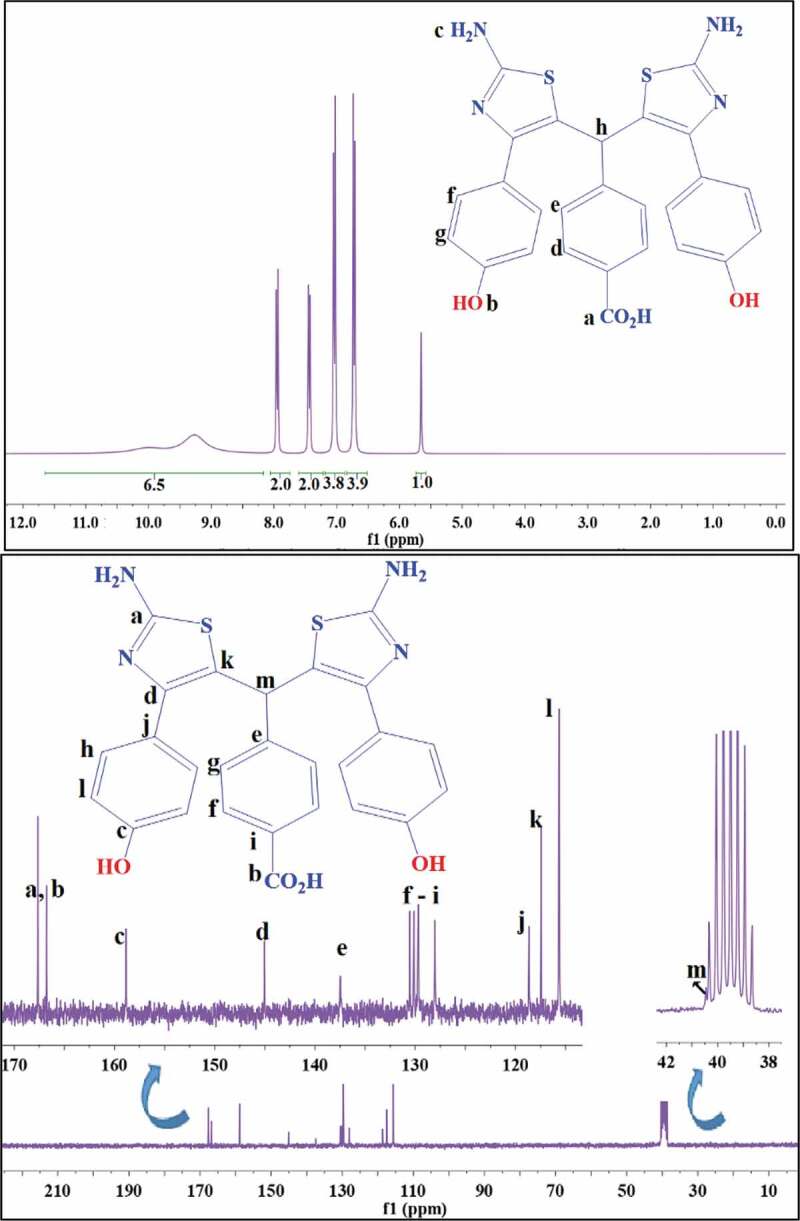
^1^ H NMR and ^13^ C NMR spectra of monomer III (top and bottom, respectively); d6-DMSO was used as solvent

The ^1^ H NMR and ^13^ C NMR spectra of monomer III are presented in Figure. 5. As can be seen in the ^1^ H NMR spectrum, eight signals are detected that are consistent with the eight hydrogens of diamine structure. Aliphatic hydrogen (h), due to the resonance of three rings, is deshielded and observed at downfield regions (δ 5.6–5.7 ppm). The resonance signals at 6.8–8.1 ppm are assigned to the aromatic protons (b, c, d, and f). The integration area of the peaks is in good agreement with the assignment. In the ^13^ C NMR spectrum of monomer III, 13 peaks were identified. The most downfield carbon peaks are attributed to the C = N of thiazole and carbonyl of carboxylic acid groups. An aliphatic carbon is observed at about 40 ppm (m) near to the solvent signals. As it is clear, the carbon and proton spectra of monomer III are well consistent with the purposed structure. The FTIR and NMR spectra of monomers I and II are provided in the supplementary data (Fig. S1).

### 3.2. Polymer synthesis

Hyperbranched polymers and dendrimers have similar properties, e.g., high solubility in organic solvents, low viscosity, and lack of significant entanglement in the solid state. The dendrimers synthesis usually needs multiple complicated deprotection and protection steps as well as a number of purification processes. In contrast, HPs are easily prepared by one-step polymerization of AB_n_ (n > 2) monomers. Herein, the new HPs were synthesized by Yamazaki method (polycondensation of AB_2_ monomers using pyridine and triphenyl phosphite). The reactions were carried out in a solution of monomers in NMP and specific amount of LiCl under N_2_ atmosphere at 100°C. Fibrous and tough products were formed when the viscous solutions of polymers were poured into the methanol. The precipitated polymer was filtrated and thoroughly washed with hot water and then with hot methanol, the yields of the resultant polymer were 89, 85 and 84 percent for HP1, HP2 and HP3, respectively. As shown in Table 2, the inherent viscosities of the prepared HPs are in the range of 0.15–0.26 dLg^−1^, which is in a similar range as reported by others [25]. This is normally consistent with this fact that the HPs, due to their globular structures, have lower inherent viscosity in comparison with linear polymers.

**Table 1.** Solubility behavior of hyperbranched polyamides (HP1-3)

Polymer	NMP	DMSO	DMAc	DMF	THF	Py	MeOH	Acetone
H-1	**++**	**++**	**++**	**++**	+ –	+ –	–	-
H-2	**++**	**++**	**++**	**++**	**+**	+	+ –	+ –
H-3	++	++	++	++	+	++	+ –	+ –

**Table 2.** Thermal behavior and inherent viscosity of HP1 HP2 and HP3

Polymer	T_g_ ^°^C	T_10_^a °^C	η^b^_Inh_ (dL/g)
HP1	113	250	0.15
HP2	172	355	0.21
HP3	205	410	0.26

**^a^**The temperature for 10% weight loss (T_10_).

**^b^**Measured at a concentration of 0.5 dL/g in DMAc at 30°C.

**Figure 6.**
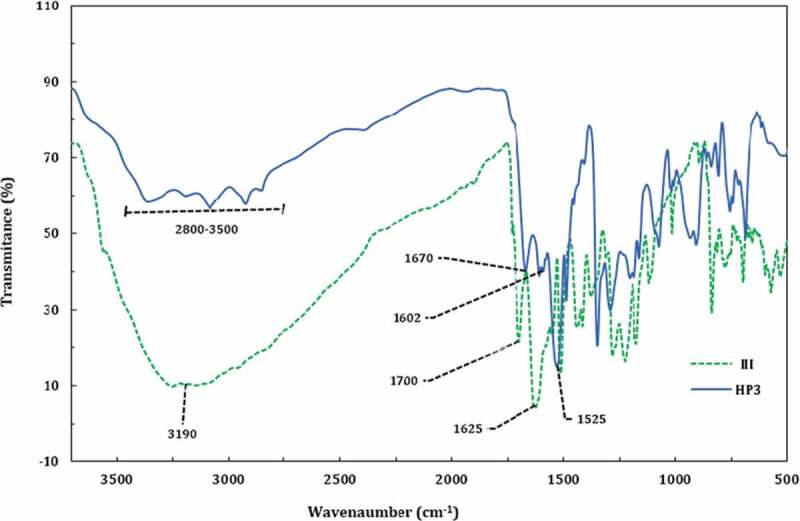
FTIR spectra of HP3 and monomer III

The structural features of these HPs were verified by IR and proton NMR spectroscopies, of which here only HP3 is presented. The HP3 exhibited the characteristic FT-IR absorption bands of the amide and terminal amine groups around 2800–3400 cm^−1^ (N-H stretch), and the absorption bands around 1670 cm^−1^ are allocated to the symmetrical stretching vibrations of carbonyl groups of amides (Figure 6). The vibration bands of the C = N of thiazole in the IR spectrum were recorded at 1602 cm^−1^. N-H bending vibration also appeared at 1525 cm^−1^. Comparing the FTIR spectrum of HP3 and its monomer shows that HP3 does not show a broad band at 2400–3400 cm^−1^ due to lack of free carboxylic acid groups and stretching absorption band at 1700 cm^−1^ of the carboxylic acid C = O in the spectrum of monomer III also is disappeared in HP3 IR spectrum.

**Figure 7.**
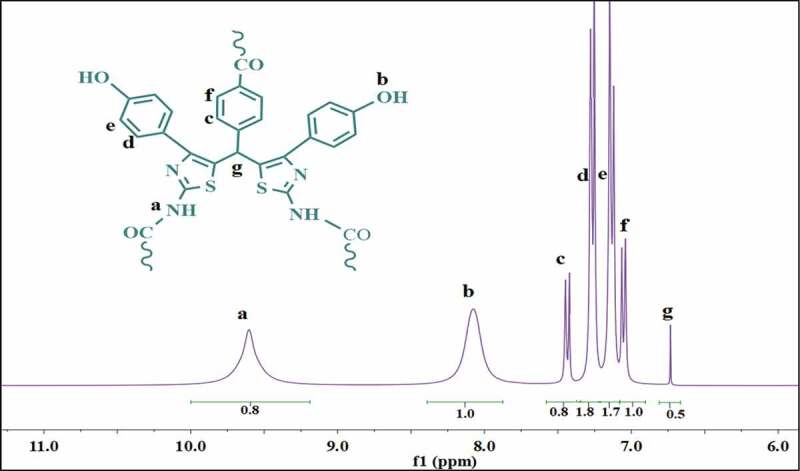
^1^ H NMR spectrum of HP3; d6-DMSO was used as solvent

The ^1^ H NMR spectra of HP3 displayed the protons of amide groups (NH) at the most downfield region, about 9.65 ppm, and the aromatic protons at the region of about 7–7.5 ppm (Figure 7).

### 3.3. Solubility of HP polymers

Herein, we investigate the solubility of HP polymers with 0.01 g of polymeric sample in 2 mL of solvent. Due to the presence of terminal amines, carboxylic acids, heterocyclic thiazole and the polar substituents in such hyperbranched structures, the solubility of HPs was expected to be very great in common organic solvents.

As is shown in Table 1, all HPs are easily soluble in aprotic polar solvents, such as N-methyl-2-pyrrolidone (NMP), dimethylacetamide (DMAc), dimethylformamide (DMF), dimethyl sulfoxide (DMSO) at room temperature, and are soluble in pyridine (Py) and tetrahydrofuran (THF), as well as partially soluble in other solvents, such as methanol and acetone at 60°C. Hydroxyl-containing HPs totally showed a better solubility in all tested solvents, while HP1 which does not have extra functional groups is a little less soluble. This trend is expectable because the strength of H-bonding between the HP3 and solvent is stronger. It can be concluded that the presence of terminal amine groups, highly branched structure, and extra polar functional groups of obtained HPs helped improve their solubility. Moreover, the presence of bulky benzene pendant groups loosens the chain packing, and subsequently, the molecules of the solvents penetrated easily into the polymers to dissolve them.

Solubility: + +: soluble at room temperature; +: soluble on heating at 60°C; + –: partially soluble on heating at 60°C; – : insoluble on heating.

### 3.4. Thermal properties

**Figure 8.**
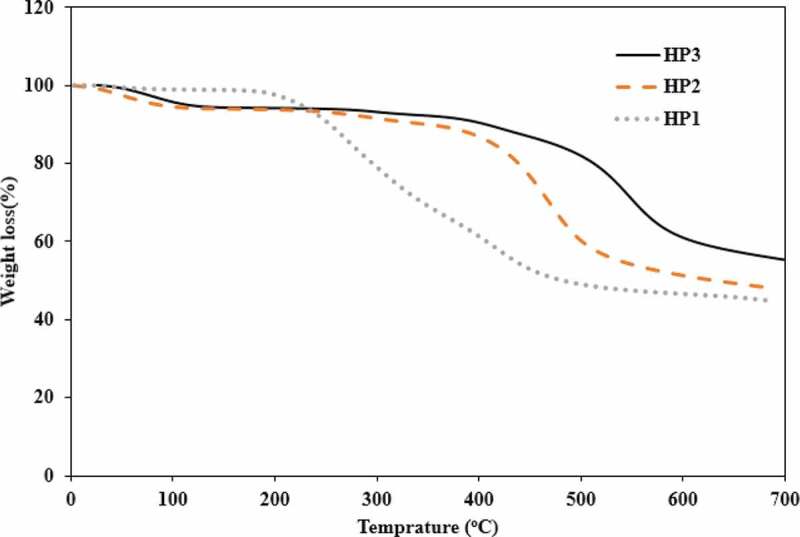
TGA curves of HP1, HP2 and HP3 at 10°C/min heating rate

Thermal properties of the HP1-3 were studied using TGA and DSC analyses. TGA analysis was carried out under an inert atmosphere. The HP1-3 thermograms are depicted in Figure 8, and the thermal characteristics are shown in Table 2. The first thermal decomposition temperatures were found to be different for HP1, HP2 and HP3. The first thermal decomposition temperature of HP3 was the highest due to its extra functional groups. For HP3 which contains hydroxyl groups, the initial thermal decomposition starts at about 500°C, and for the nitro-containing polymer, it starts at near 380°C, while for HP1 which does not have any extra functional groups the initial decomposition starts at about 230°C. This behavior can be related to the lower molecular weight of HP1. As seen in Table 2, HP3 and HP1 have the highest (0.26) and lowest (0.15) inherent viscosity, respectively. Moreover, the initial small weight loss around 100°C for both HP2 and HP3 is due to trapped water molecules in their structures. HP2 and HP3 have extra functional groups which make them apt to absorb humidity easily.

Also, the remaining weights for HPs at 700°C ranged from 45% to 56% in N_2_ atmosphere. The temperature for 10% weight loss (T_10_) is usually used as an important criterion to determine the polymer thermal stability. The T_10_ values of HPs determined from their original thermograms are tabulated in Table 2. They are in the range of 255–410°C in N_2_ atmosphere. According to these data, it can be concluded that the incorporation of the functional groups into the structure of HPs improves their thermal stability and solubility mechanism because of increasing their molecular weight.

One of the most important and useful features of HPs is their T_g_ arisen from amorphous structure which has been reported for most of them [26]. For linear polymers, the T_g_ is related to the large segmental motions in the polymer chain segments and the end groups role can be ignored above a certain molecular weight. Since the segmental motions of the HPs are influenced by the branching points and there are a lot of end groups, their effect on T_g_ is more difficult to neglect. It has been stated that the HP glass transition is a translational movement of the entire molecule instead of a segmental movement [27]. Moreover, the results reported by Viot [28] showed that end group structures and polarity have a large impact on the T_g_. For instance, the T_g_ values of the hyperbranched aromatic polyesters with different end groups are different and are shifted about 100 °C (from 250 to 150 °C) through changing carboxylic acid to acetate end groups.

The T_g_ values of the HPs, summarized in Table 2, were obtained by DSC measurements at 10°C/min heating rate under N_2_. The exact value of T_g_ was determined from the second heating run of DSC measurements. The T_g_ values of the polymers were found to be between 113 and 205 ^°^C which were improved by the presence of extra functional groups (–NO_2_ and – OH).

Glass transition temperature directly depends on the molecular weight. This dependence becomes vital at lower molecular weights owing to the free volume around the chain ends [29]. HP1 showed the lowest T_g_ value (113°C) among three HPs, which could be attributed to its low molecular weight (the lowest inherent viscosities). Moreover, HP3 with para-substituted hydroxyl groups exhibited the highest T_g_ of 205 ^o^C, whereas a medium value of 172 ^o^C was observed for HP2, which contains nitro groups. Thus, it can be concluded that the incorporation of extra functional groups into the HP chain serves to enhance its thermal stability.

Particularly, the presence of hydroxyl groups significantly increases the solubility of growing polymer chains, which helps further the growth of polymer chain lengths and consequently improves the thermal properties.

### 3.5. Fluorescent study

**Figure 9.**
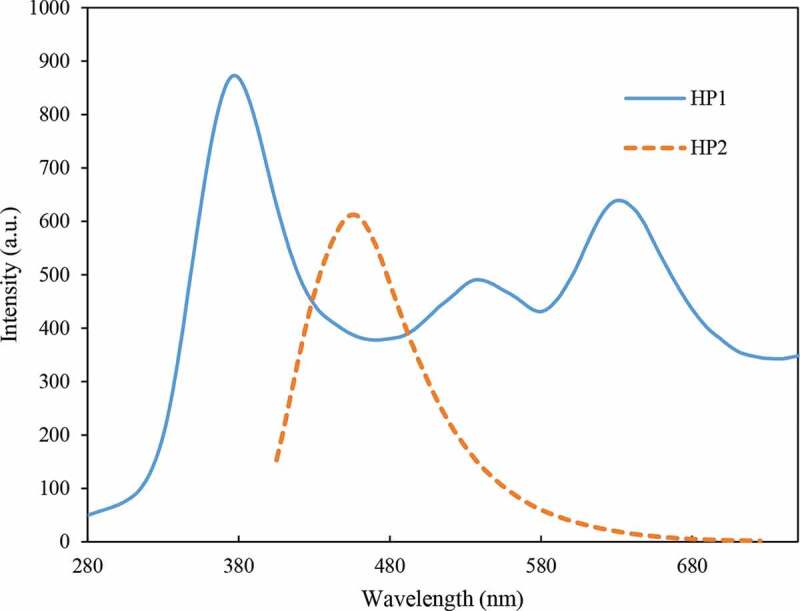
PL emission of HP1 and HP2

Thiazole heterocycles have been shown to be strong fluorescent compounds [30]. Moreover, it has been proved that thiazole rings act as effective nonlinear optical and photochromic materials [31,32]. Therefore, the fluorescence properties of the prepared hyperbranched polymers were studied with the solution of HPs in DMSO. As seen in Figure 9, HP1 illustrates three maximum emission peaks at 380, 540, and 635 nm that can be attributed to the thiazole and benzene rings. But the nitro-containing HP3 just shows one emission peak around 460 nm, which is attributed to the electron withdrawing nature of nitro groups which cause merging of emission peaks of HP1, but this is not the case with HP3. This HP does not show any fluorescent properties, which probably is due to the excited state intramolecular proton transfer (ESIPT) between the NH/OH- and N-containing groups of thiazole rings, leading to fluorescence quenching in OH-containing HPs [33].

## 4. Conclusion

This study introduced new hyperbranched polyamides (HP1, HP2 and HP3) containing different functional groups and thiazole ring. HPs were successfully synthesized through direct polycondensation reaction of novel AB_2_ monomer. Novel HPs were characterized using IR and NMR spectroscopic means. The thermal stability and solubility of the HP2 and HP3 which contain extra functional groups were compared to those of HP1, the hyperbranched polyamide without functional groups. Extra functional groups, due to providing better solubility of growing polymer chains in polymerization step, lead to higher molecular weight and better thermal stability. The optical properties of prepared HPs were investigated using fluorescence analysis. The fluorescence analysis showed that in DMSO as an aprotic polar solvent, HP1 has good fluorescence properties, while HP2 showed just one emission peak and HP3 does not have any fluorescence properties. A decrease in fluorescence properties is a result of an increase in the inter- and intra-molecular interactions. HP2 and HP3 exhibited superior solubility and good thermal properties in common organic solvents which make them useful for different applications, such as polymer modifying agents and nanomaterials supports. In our future research, we will report advanced applications for HP2 and HP3 separately.
